# Supplementary material for: A renal clearable fluorogenic probe for in vivo β-galactosidase activity detection during aging and senolysis
Source: Nat Commun. 2024 Jan 26;15:775. doi: 10.1038/s41467-024-44903-1 (PMC10817927; doi:10.1038/s41467-024-44903-1)
Supplement: Supplementary file 1 — Supplementary Information [file 41467_2024_44903_MOESM1_ESM.pdf]

Supplementary Information

A renal clearable fluorogenic probe for *in vivo* β-galactosidase activity detection during aging and senolysis

Sara Rojas-Vázquez[1,2,3*], Beatriz Lozano-Torres[1,2,4*], Alba García-Fernández[1,2,4], Irene Galiana[1,2,4,5], Ana Perez-Villalba[6,7], Pablo Martí-Rodrigo[3,7], M. José Palop[3,7], Marcia Domínguez[1,2], Mar Orzáez[4,8],Félix Sancenón[1,2,4,5], Juan F. Blandez[1,2,4], Isabel Fariñas[3,7] and Ramón Martínez-Máñez[1,2,4,5]

[1]Instituto Interuniversitario de Investigación de Reconocimiento Molecular y Desarrollo Tecnológico (IDM), Universitat Politècnica de València- Universitat de València, Spain.

[2]CIBER de Bioingeniería, Biomateriales y Nanomedicina (CIBER-BBN), Spain.

[3]Instituto de Biotecnología y Biomedicina (BIOTECMED), Universitat de València, Spain.

[4]Unidad Mixta UPV-CIPF de Investigación en Mecanismos de Enfermedades y Nanomedicina, Universitat Politècnica de València, Centro de Investigación Príncipe Felipe, Valencia, Spain.

[5]Unidad Mixta de Investigación en Nanomedicina y Sensores. Universitat Politècnica de València, IIS La Fe, Valencia, Spain.

[6]Laboratory of Animal Behavior Phenotype (L.A.B.P.). Facultad de Psicología. Universidad Católica de Valencia, Spain.

[7]Centro de Investigación Biomédica en Red de Enfermedades Neurodegenerativas (CIBERNED), Spain.

[8]Centro de Investigación Príncipe Felipe, Valencia, Spain.

* Equal contribution

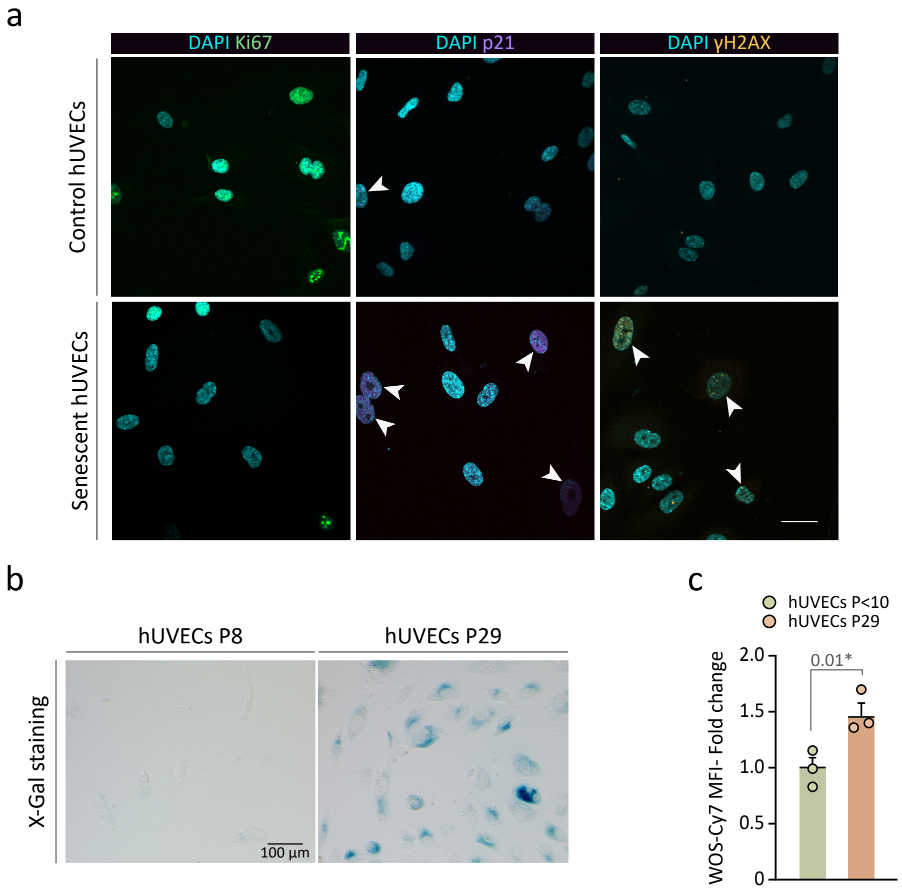

Supplementary Figure 1. Characterisation of palbociclib-induced senescence in hUVECs and detection of SA-β-Gal activity during replicative senescence in these cells. a, Representative confocal images of control and senescent hUVECs labeled with the proliferation marker Ki67 and the senescence-associated markers p21 and γH2AX. The arrows indicate immune-positive cells in which fluorescence signal is not easily discernible. **b**, X-Gal histochemical staining in hUVECs after being passaged 29 *vs.* 8 times. **c,** Detection of β-Gal activity in hUVECs passaged either less than 10 times or 29 times by quantifying WOS-Cy7Gal-associated fluorescence using flow cytometry. The fold change refers to hUVECs that were passaged fewer than 10 times. Paired two-tailed Student's t-test statistical analysis was used to evaluate the detection of SA-β-Gal activity with WOS-Cy7Gal in the model of replicative senescence with hUVECs. Scale bar: 100 µm.

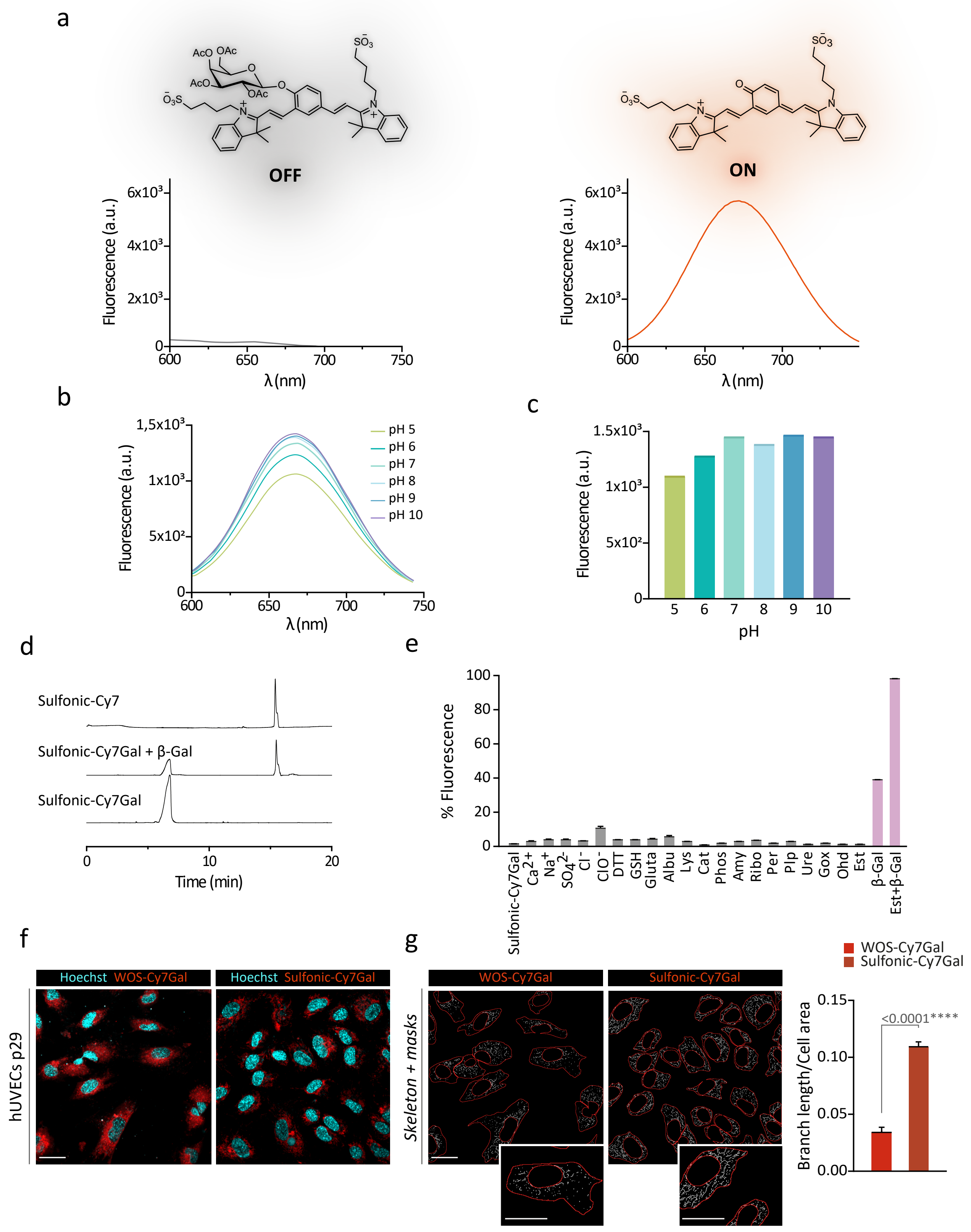

Supplementary Figure 2. Sulfonic-Cy7Gal fluorescence emission relies on β-Gal activity. a, Fluorescence emission spectra (λexc = 580 nm) of sulfonic-Cy7Gal (left) and sulfonic-Cy7 (right) in

aqueous solutions at pH 7. **b**, Fluorescence spectra (λex = 580 nm) of sulfonic-Cy7 (10^{-5} M) H_2O at pH 5, 6, 7, 8, 9 and 10. **c**, Emission intensity at 665 nm (λex = 580 nm) of sulfonic-Cy7 (10^{-5} M) H_2O solutions at pH 5, 6, 7 8, 9 and 10. **d**, Chromatograms of sulfonic-Cy7, sulfonic-Cy7Gal + β-Gal and sulfonic-Cy7Gal. For hydrolysis studies, aqueous solutions of sulfonic-Cy7 and sulfonic-Cy7Gal at a concentration of 10^{-5} M (pH 7) were prepared. Human β-Gal was then added (5 µl) to sulfonic-Cy7Gal solutions and chromatograms were acquired after 30 min (λabs = 358 nm for sulfonic-Cy7Gal and λabs = 254 nm for sulfonic-Cy7) with a Waters 1525 binary HPLC pump equipped with a Waters 2990 diode array detector. Chromatograms were obtained using Empower 3 software. Conditions: Kromasil-C18 column, 0.8 ml/min, H_2O:MeOH gradient elution: 90:10 to 10:90. Data analysis was performed using OriginPro8 software. **e**, Fluorescence intensity changes of sulfonic-Cy7Gal solutions (20 µM) treated with β-Gal and with interfering species (n=3). From left to right: Blank (only sulfonic-Cy7Gal, 20 µM), Ca^{2+}, Na^{+}, SO_4^{2-}, Cl^{-}, ClO^{-}, DTT (DL-dithiothreitol), GSH (γ-L-glutamyl-L-cysteinyl-glycine), Glu (glutamate), Albu (albumin from human serum), Lys (lysozyme from chicken egg white), Cat (catalase from bovine liver), Phos (phosphatase Alkaline from porcine kidney), Amy (alfa-amylase from porcine pancreas), Ribo (ribonuclease A from bovine pancreas), Per (peroxidase from horseradish), Plp (Phosphorylase a from rabbit muscle), Ure (urease from Canavalia ensiformis (Jack bean) Type III), Gox (glucose oxidase from Aspergillus niger), OHd (alcohol dehydrogenase from Saccharomyces cerevisiae), Est (esterase from porcine liver), β-Gal (human β-Gal). Cations (150 µM), anions (150 µM), amino acids (150 µM), proteins (150 µg/mL) and enzymes (150 µg/mL). All measurements were acquired at 37 °C for 0.5 h. **f**, Confocal images comparing WOS-Cy7Gal and sulfonic-Cy7Gal signal patterns in hUVECs passaged 29 times. Note that while WOS-Cy7Gal displayed a dotted pattern, sulfonic-Cy7Gal showed a more diffuse one. **g**, Images of skeletonized masks for each probe and quantification of branch length normalized by cell cytoplasm area. At least 30 cells were considered in this analysis. The graphs **e** and **g** show the mean ± SEM. For statistical analysis of the signal patterns of the WOS-Cy7Gal and sulfonic-Cy7Gal probes in the **g**-graph, an unpaired two-tailed Student's t-test was used. Scale bars: 100 µm.

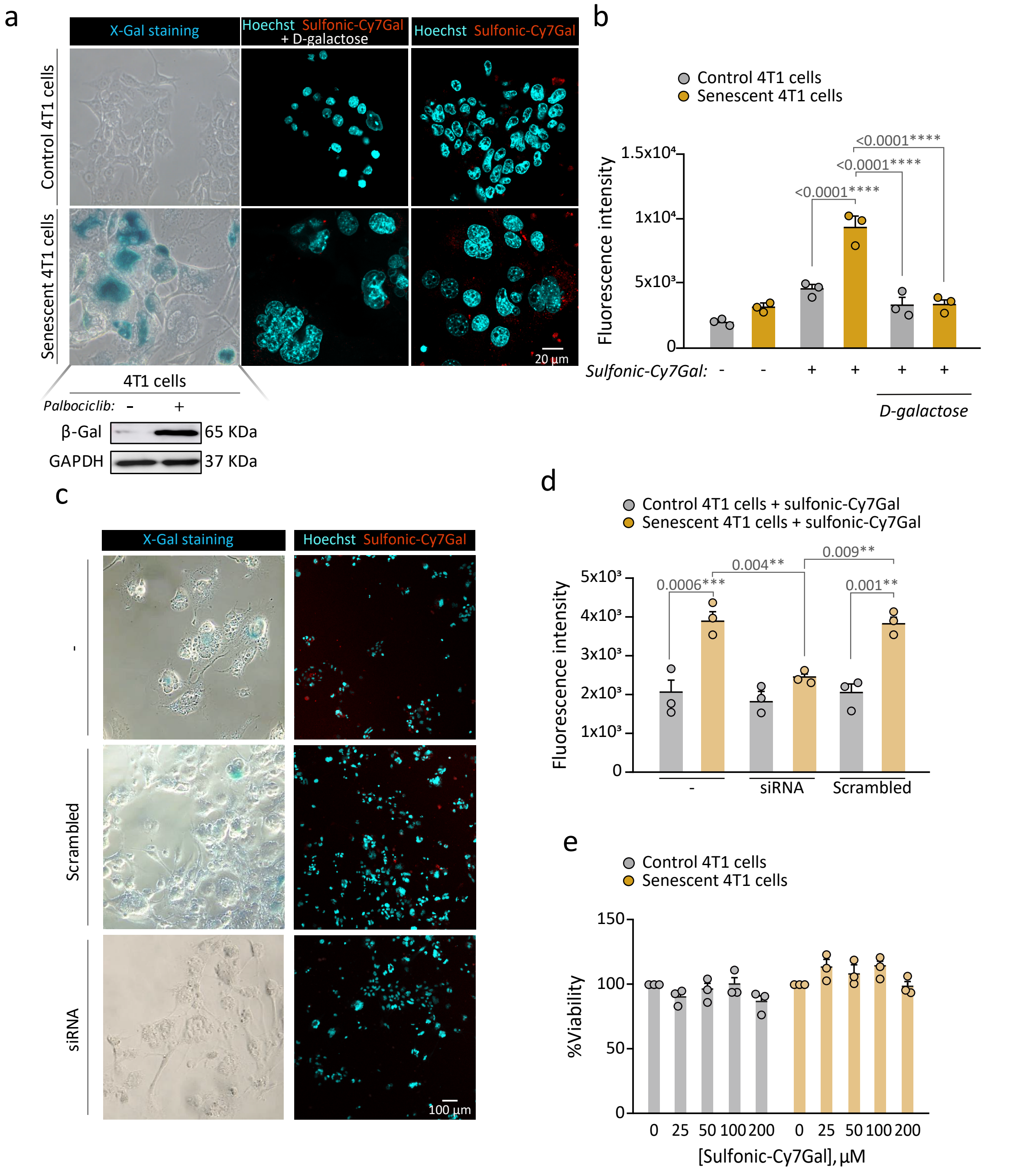

Supplementary Figure 3. Sulfonic-Cy7Gal selectively detects β-Gal activity without cytotoxic effects. **a**, X-Gal histochemistry for SA-β-Gal activity evaluation in control 4T1 cells (top) and palbociclib-treated 4T1 cells (bottom) and determination of β-Gal protein expression by western-blot. Confocal images comparing control 4T1 cells to those treated with palbociclib in the presence of 20 µM sulfonic-Cy7Gal, with and without pre-treatment using a β-Gal enzyme inhibitor (D-galactose). **b**, Quantification of sulfonic-Cy7 dye fluorescence emission intensity from confocal microscopy images of control and palbociclib-treated 4T1 cells. Fluorescence assessment was performed in the absence and presence of 20 µM sulfonic-Cy7Gal, without pre-treating the cells or after pre-treating them with D-galactose. **c**, X-Gal histochemical reaction and confocal imaging with the sulfonic-Cy7Gal probe for the

evaluation of SA-β-Gal activity in 4T1 cells treated with palbociclib and exposed to siRNA, or scrambled for the *Glb1* gene (encoding the β-Gal enzyme), compared to unexposed (-) cells. **d**, Quantification of fluorescence emission intensity associated with sulfonic-Cy7Gal from confocal images as shown in **c**. **e**, Viability assay (cellTiter-Glo®) of control and senescent 4T1 cells in the presence of sulfonic-Cy7Gal. The graphs show the mean ± SEM. The number of independent biological samples (represented as dots) used are indicated in the graphs with the exception of **e** (n=3). One-way ANOVA and Tukey's post-hoc multiple comparison test were used for statistical analysis. Scale bars: **a** 20 μm, **c** 100 μm.

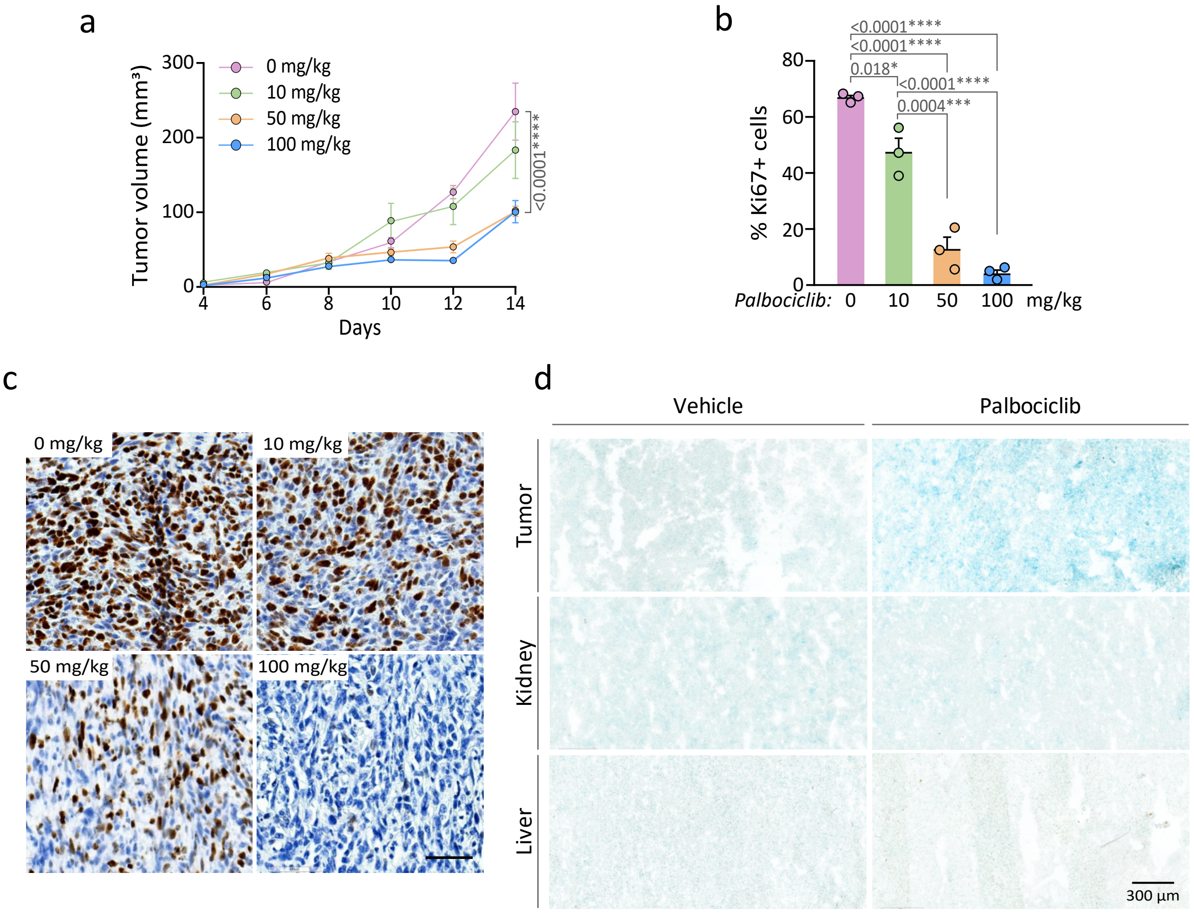

Supplementary Figure 4. Chemotherapy-induction of senescence *in vivo* in a breast cancer animal model. **a**, BALB/cByJ female mice carrying orthotopic 4T1 mammary tumors were treated daily with different doses of palbociclib (oral gavage: 0, 10, 50 or 100 mg/kg). For each tumor, the relative volume change was calculated as V = (a x b²)/2 where a is the longest and b is the shortest of two perpendicular diameters. The graph represents the tumor volume over time (14 days) in palbociclib-treated *vs.* non-treated mice bearing 4T1 tumors. **b**, Quantification of immunohistochemical detection of Ki67 in paraffin sections of tumors. Note that the number of Ki67 positive cells (proliferative cells) decreases as the dose of palbociclib increases. **c**, Images of immunohistochemical detection of Ki67 in paraffin sections of tumors from mice treated with different doses of palbociclib (labeled in the images). **d**, X-Gal staining for SA-β-Gal activity assessment comparing tumors with other organs such as kidneys and liver in vehicle (untreated) or palbociclib-treated mice (100 mg/kg). The graphs show the mean ± SEM. The number of independent biological samples (represented as dots) used are indicated in the graphs. Two-way ANOVA and one-way ANOVA, followed by Tukey's post-hoc multiple comparison test, were used for statistical analysis of graph **a** and **b**, respectively. Scale bars: **c** 100 µm, **d** 300 µm.

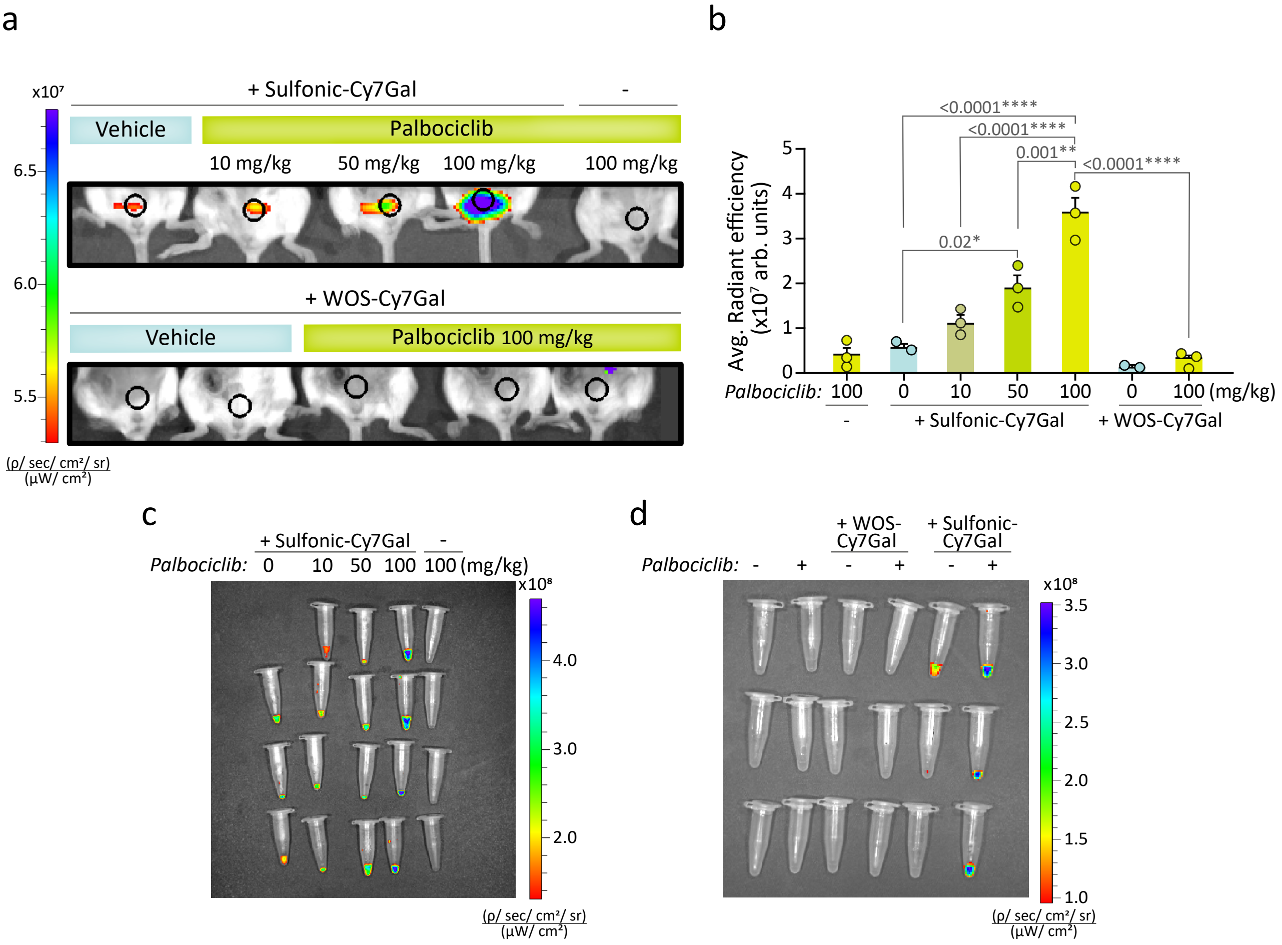

Supplementary Figure 5. Sulfonic-Cy7Gal monitors palbociclib treatment in mice bearing breast 4T1 tumors. a, Representative IVIS® images of BALB/cByJ mice with breast tumors treated with different doses of the senescence-inducing drug palbociclib and injected with sulfonic-Cy7Gal or WOS-Cy7Gal, specifically showing the lower urinary tract. The images showcase ROIs delimiting the bladder area. **b**, Fluorescence (average radiant efficiency) quantification of these ROIs. **c**, IVIS® imaging of urine samples from mice bearing mammary tumors, treated with increasing doses of palbociclib and injected with sulfonic-Cy7Gal. Observe that the highest fluorescent levels in urine correspond to the maximum dose of palbociclib, while non-injected mice treated with the same dose do not emit any fluorescent signal. **d**, IVIS® imaging of urine samples from palbociclib-treated (100 mg/kg) or untreated mice bearing mammary tumors, and injected with WOS-/sulfonic-Cy7Gal or vehicle (DMEM). The graph shows the mean ± SEM. The number of independent biological samples (represented as dots) used are indicated in the graph. One-way ANOVA and Tukey's post-hoc multiple comparison test were used for statistical analysis.

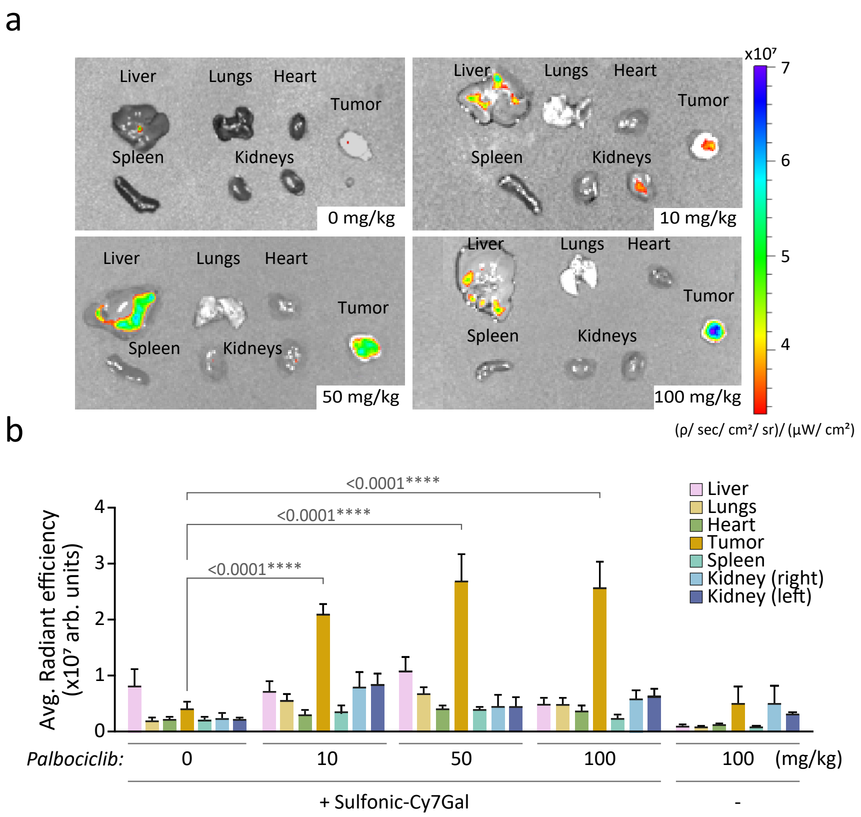

Supplementary Figure 6. *Ex vivo* analysis of sulfonic-Cy7Gal in different organs from mice bearing mammary tumors and treated with palbociclib. **a**, *Ex vivo* IVIS® imaging of a variety of organs (liver, lungs, heart, spleen, kidneys, tumors) from mice bearing orthotopic 4T1 tumors, treated with different doses of palbociclib (0, 10, 50, 100 mg/kg) and injected with sulfonic-Cy7Gal. **b**, Fluorescence (average radiant efficiency) readout of ROIs delimiting each organ in the conditions exposed in image **e**, compared to palbociclib-treated mice (100 mg/kg) that were not injected with the probe. The graphs values show mean ± SEM. The number of independent biological samples in graph **b** is n=3. Two-way ANOVA and Tukey's post-hoc multiple comparison test were used for statistical analysis.

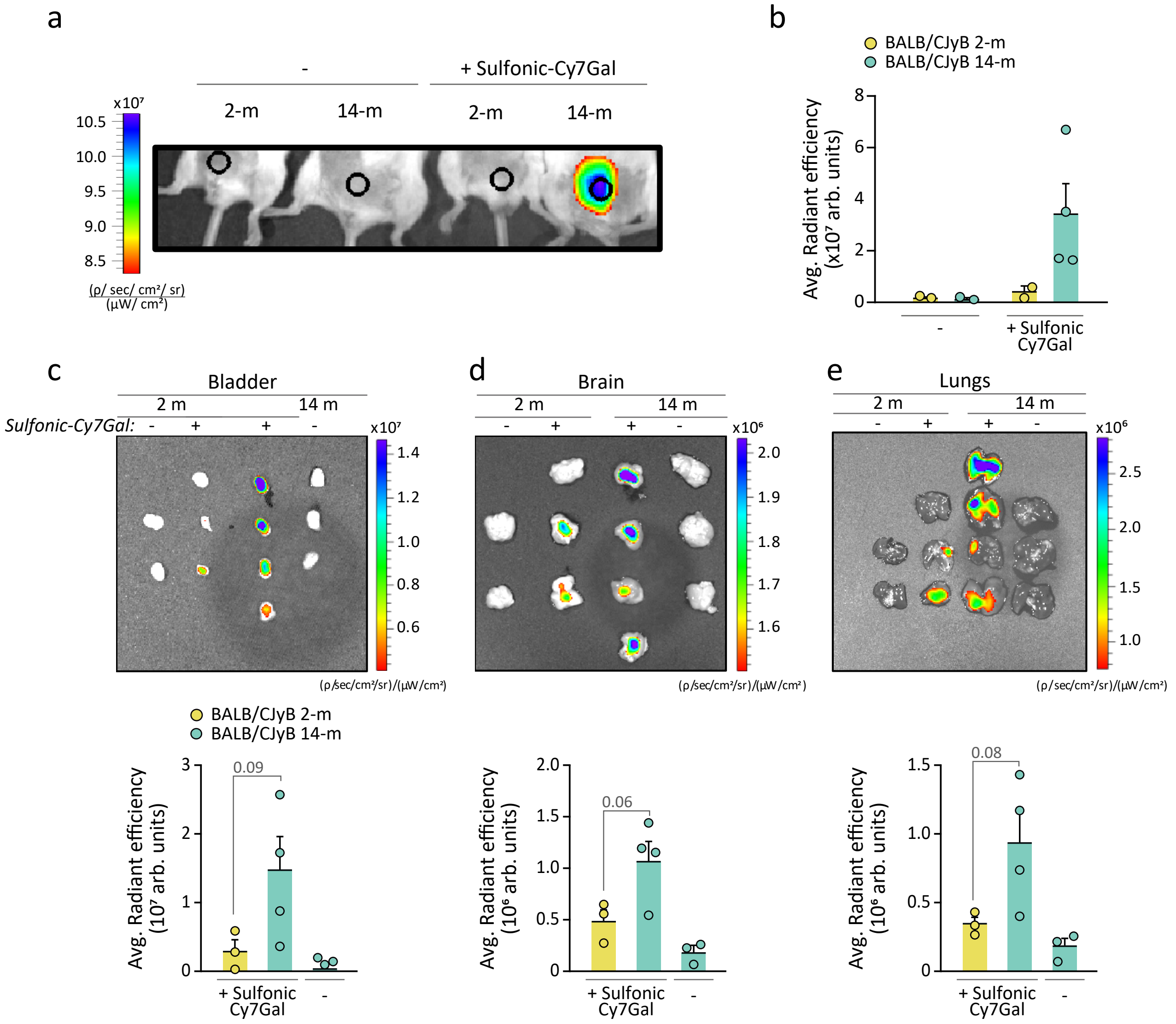

Supplementary Figure 7. An insight into sulfonic-Cy7Gal biodistribution in young *vs.* old BALB/cByJ mice. **a**, *In vivo* IVIS® imaging of BALB/cByJ mice of 2-m and 14-m injected or not with sulfonic-Cy7Gal, specifically showing the lower urinary tract and excluding the injection site. The images showcase ROIs delimiting the bladder area. **b**, Fluorescence (average radiant efficiency) quantification of these ROIs. **c**, *Ex vivo* IVIS® images of the bladder of 2- and 14-m BALB/cByJ mice that were injected i.p. or not with sulfonic-Cy7Gal and measurement of the probe-associated fluorescence in this organ. **d**, *Ex vivo* IVIS® images of the brain of 2- and 14-m BALB/cByJ mice that were injected i.p. or not with sulfonic-Cy7Gal, and readout of the probe-related fluorescence in this organ. **e**, *Ex vivo* IVIS® images of the lungs of 2- and 14-m BALB/cByJ mice that were injected i.p. or not with sulfonic-Cy7Gal and probe-related fluorescence readout in this organ. The graphs show the mean ± SEM. The number of independent biological samples (represented as dots) used and the exact p-values are indicated in the graphs. Unpaired Student's two-tailed t-test was used for statistical analysis.

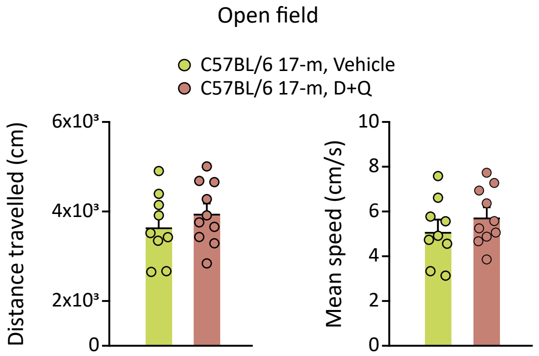

Supplementary Figure 8. Locomotion assessment after senolysis. Locomotion assessment of C57BL/6 mice after senolytic treatment with D+Q during the open field test. Observe that there are no differences at this level between treated and untreated C57BL/6 mice. The graph shows the mean ± SEM. The number of independent biological samples (represented as dots) used are indicated in the graphs. Statistical analysis with unpaired two-tailed Student's t-test showed no significative differences between both groups (p-value>0.05).

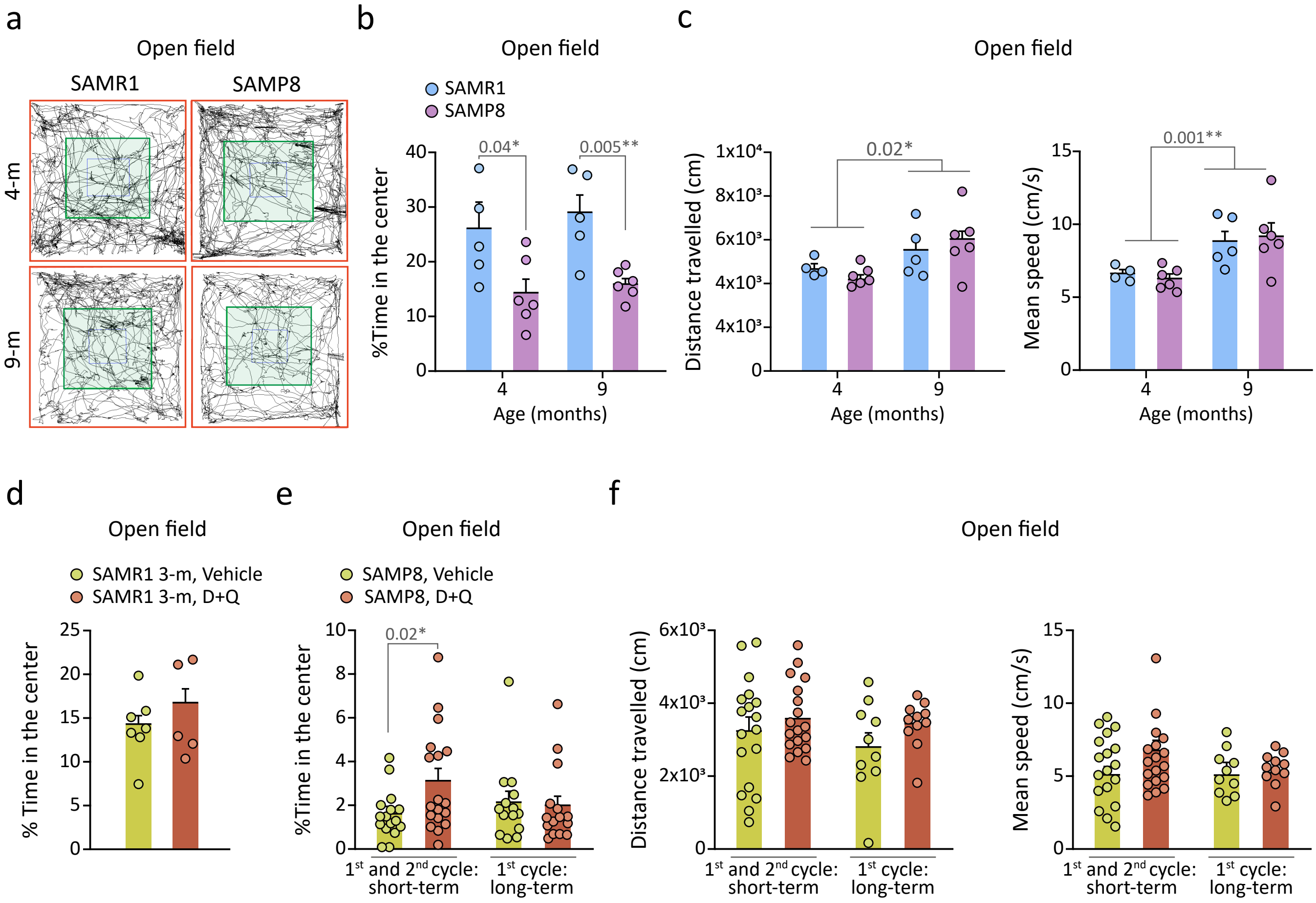

Supplementary Figure 9. Anxious behavior and locomotion assessment during accelerated aging and after senolysis. **a**, Representative map of movement in the open field test comparing 4- and 9-m SAMR1 to SAMP8 mice. **b**, Percentage of time that 4- and 9-m SAMR1 and SAMP8 mice spent in the center of the open field (green area). **c**, Locomotion assessment of 4- and 9-m SAMR1 and SAMP8 in the open field. Notice that differences in avoidance of the center area in the two genetic backgrounds are not due to differences in locomotion. **d**, Percentage of time 3-m SAMR1 mice spent in the center of the open field after 3 days receiving D+Q or vehicle orally. Note that D+Q senolytic drugs per se do not exhibit an anxiolytic effect. **e**, Percentage of time SAMP8 mice, treated with D+Q or vehicle, spend in the central area of the open field. Notice that, only when we assessed short-term anxious behavior after senolytic treatment, a significant improvement in open field test performance (decreased anxiety) was observed in mice treated with D+Q. **f**, Evaluation of locomotion-associated parameters (mouse total distance travelled and mean speed, in the open field test) in SAMP8 mice after senolytic treatment. Note that locomotion is not affected by the treatment, either when assessed in the short- or long-term after the senolytic intervention. The graphs show the mean ± SEM. The number of independent biological samples (represented as dots) used and the exact p-values are indicated in the graphs.Unpaired two-tailed t-test was used to analyze mice anxious behavior (**b**, **d**, **e**) in the open field test, while locomotor behavior was analyzed with a two-way ANOVA comparing simultaneously SAM background x age (**c**), as well as, SAMP8 D+Q/vehicle x treatment evaluation term (**f**).
