# Supplementary material for: A renal clearable fluorogenic probe for in vivo β-galactosidase activity detection during aging and senolysis
Source: Nat Commun. 2024 Jan 26;15:775. doi: 10.1038/s41467-024-44903-1 (PMC10817927; doi:10.1038/s41467-024-44903-1)
Supplement: Supplementary file 3 — Reporting Summary [file 41467_2024_44903_MOESM3_ESM.pdf]

nature portfolio

Corresponding author(s): Ramón Martínez-Máñez and Isabel Fariñas

Last updated by author(s): Dec 15, 2023

Reporting Summary

Nature Portfolio wishes to improve the reproducibility of the work that we publish. This form provides structure for consistency and transparency in reporting. For further information on Nature Portfolio policies, see our Editorial Policies and the Editorial Policy Checklist.

Statistics

For all statistical analyses, confirm that the following items are present in the figure legend, table legend, main text, or Methods section.

n/a	Confirmed	
☐	☒	The exact sample size (*n*) for each experimental group/condition, given as a discrete number and unit of measurement
☐	☒	A statement on whether measurements were taken from distinct samples or whether the same sample was measured repeatedly
☐	☒	The statistical test(s) used AND whether they are one- or two-sided<br>*Only common tests should be described solely by name; describe more complex techniques in the Methods section.*
☒	☐	A description of all covariates tested
☐	☒	A description of any assumptions or corrections, such as tests of normality and adjustment for multiple comparisons
☐	☒	A full description of the statistical parameters including central tendency (e.g. means) or other basic estimates (e.g. regression coefficient) AND variation (e.g. standard deviation) or associated estimates of uncertainty (e.g. confidence intervals)
☐	☒	For null hypothesis testing, the test statistic (e.g. F, t, r) with confidence intervals, effect sizes, degrees of freedom and P value noted<br>*Give P values as exact values whenever suitable.*
☒	☐	For Bayesian analysis, information on the choice of priors and Markov chain Monte Carlo settings
☒	☐	For hierarchical and complex designs, identification of the appropriate level for tests and full reporting of outcomes
☒	☐	Estimates of effect sizes (e.g. Cohen's *d*, Pearson's *r*), indicating how they were calculated

Our web collection on statistics for biologists contains articles on many of the points above.

Software and code

Policy information about availability of computer code

Data collection	Excel Microsoft Office Proffesional plus 2021.
Data analysis	For flow cytometry analysis FlowJo_v10.8.1 software was used. Statistical analysis was performed with GraphPad 5.0 (Prism) software. Live Imaging software from Caliper Life Sciences was used for IVIS-spectrum imaging analysis. FluorEssence™ for Windows software was used for fluorescence measurements in urine.

For manuscripts utilizing custom algorithms or software that are central to the research but not yet described in published literature, software must be made available to editors and reviewers. We strongly encourage code deposition in a community repository (e.g. GitHub). See the Nature Portfolio guidelines for submitting code & software for further information.

Data

Policy information about availability of data

All manuscripts must include a data availability statement. This statement should provide the following information, where applicable:

- Accession codes, unique identifiers, or web links for publicly available datasets
- A description of any restrictions on data availability
- For clinical datasets or third party data, please ensure that the statement adheres to our policy

The authors declare that the data supporting the findings of this study are available within the manuscrit and its supplementary information files, as well as in Source Data File.

Human research participants

Policy information about studies involving human research participants and Sex and Gender in Research.

Reporting on sex and gender	Not applicable.
Population characteristics	Not applicable.
Recruitment	Not applicable.
Ethics oversight	Not applicable.

Note that full information on the approval of the study protocol must also be provided in the manuscript.

Field-specific reporting

Please select the one below that is the best fit for your research. If you are not sure, read the appropriate sections before making your selection.

☒ Life sciences ☐ Behavioural & social sciences ☐ Ecological, evolutionary & environmental sciences

For a reference copy of the document with all sections, see nature.com/documents/nr-reporting-summary-flat.pdf

Life sciences study design

All studies must disclose on these points even when the disclosure is negative.

Sample size	Most of the study has been carried out using different mouse strains (BALB/cByJ, C57BL/6, SAMP8 and SAMR1). The sample size in the experiments has never been less than three specimens per condition/genotype/age/measurement. From a strictly statistical point of view, a larger number of samples is always desirable, but the experience of different researchers in this field, and the literature review of other works, suggests that our sampling size should be entirely adequate when working with mouse strains. Therefore, we chose our sample size based on previous experience and the literature. We have indicated the sample size ("n") in each figure and/or figure legend and represented independent values as dots in addition to the mean and the standard error of the mean.
Data exclusions	The Rout method (Q=5%), available in GraphPad Prism 8.0 statistical software, was used to identify and exclude outliers. Only 4 outliers were identified pertaining to the measurement of sulfonic-Cy7Gal-associated fluorescence in urine samples from SAMP8 mice treated with senolytics.
Replication	All animal experiments have been performed more than once, either during the analysis or during the review phase of the previous submission. In addition, the senolytic treatments and their evaluation in SAMP8 and C57BL/6 mice were first implemented in a pilot study with a small cohort of animals. Therefore, all results are reproducible.
Randomization	No randomization was used. Instead, stratification was used with respect to sex and age.
Blinding	For all in vivo experiments performed by various collaborators, there was always someone who only knew the genotype condition and others the treatment condition or did not know both, thus giving a fully equivalent management to all mice and samples. Several investigators performed in vitro studies but without blind conditions as senescent cells with the probe were compared to senescent cells without it and the same for control cells.

Reporting for specific materials, systems and methods

We require information from authors about some types of materials, experimental systems and methods used in many studies. Here, indicate whether each material, system or method listed is relevant to your study. If you are not sure if a list item applies to your research, read the appropriate section before selecting a response.

Materials & experimental systems

n/a	Involved in the study
☐	☒ Antibodies
☐	☒ Eukaryotic cell lines
☒	☐ Palaeontology and archaeology
☐	☒ Animals and other organisms
☒	☐ Clinical data
☒	☐ Dual use research of concern

Methods

n/a	Involved in the study
☒	☐ ChIP-seq
☐	☒ Flow cytometry
☒	☐ MRI-based neuroimaging

Antibodies

Antibodies used	CD31 (BD, cat. no. 740239, clone 390, lot 1306154, dilution 1:100); CD45 (BD, cat. no. 563890, clone 30-F11, lot 9170854, dilution 1:100); p16 (Abcam, cat. no. ab211542, clone EPR20418, lot GR3319822-1,2; dilution 1:100); Lamin B1 (Abcam, cat. no. Ab16048, polyclonal, lot GR3398323-1, dilution 1:100); Ki67 (Abcam, cat. no. ab15580, polyclonal, lot GR3293897-2, dilution 1:50 and Cell Signaling, cat. no. 9129T, D3B5, dilution 1:800); p21 (Abcam, cat. no. ab109520; clone EPR362, dilution 1:100); phospho-histone-H2AX (ser 139) (Millipore, cat. no. 05-636-I, clone JBW301, dilution 1:100); GAPDH (Cell Signaling, cat. no. 2118; clone 14C10, dilution 1:3000); Beta-gal (Cell Signaling, cat. no. clone E2U2, dilution 1:1000); Alexa 647-donkey anti-rabbit (Invitrogen, cat. no. A31573, polyclonal, lot 3668215); Alexa 488-donkey anti-rabbit (Molecular Probes, cat. no. A21206, dilution 1:600); Cy3-donkey anti-mouse (Jackson Immuno-Research, cat. no. 715-165-151, dilution 1:600).
Validation	- Anti-CD31: endothelial cells marker; Famiglietti J et al. J Cell Biol. (1997) 138(6):1425-1435.<br>- Anti-CD45: immune cells marker; Simon DI et al. J Clin Pathol. (2000) 105(3):293-300.<br>- Anti-p16: cell cycle inhibitor marker; Xiang QY et al. Aging. (2020) 12:26080-26094.<br>- Anti-Lamin B1: nuclear lamina B marker (reduced protein levels in senescent cells); Yang D et al. Am J Cancer Res. (2021) 11:370-388.<br>- Anti-Ki67: cell proliferation marker; Marqués-Torrejón et al., Cell Stem Cell (2013) 12:88.<br>- Anti-p21: cell cycle inhibitor marker; Liu L et al. Mol Med Rep. (2021) 24:N/A.<br>- Anti-phospho-histone H2AX: DNA damage foci marker; Tanaka T et al. Methods mol biol. (2009) 523:161-168.<br>- Anti-GAPDH: western-blot loading control; Calbay O et al. iScience (2023) 26(12): 108408.<br>- Anti-beta-gal: lysosomal enzyme; Lee BY et al. Aging Cell (2006) 5: 187-195.<br>- Alexa 647-donkey anti-rabbit: secondary antibody; Belenguer G et al. Cell Stem Cell. (2021) 28(2):285-299.<br>- Alexa 488-donkey anti-rabbit: secondary antibody; Holmberg et al., J. Biol. Chem. (2002) 277:31918.<br>-Cy3-donkey anti-mouse: secondary antibody; Ferrón et al., Nature (2011) 475:381.

Eukaryotic cell lines

Policy information about cell lines and Sex and Gender in Research

Cell line source(s)	4T1 mouse mammary tumour cell line was obtained from the American Type Culture Collection (ATCC) and primary endothelial cells from neonatal human (indifferent sex) umbilical cord (hUVECs) were obtained from Sigma-Aldrich (SCCE001).
Authentication	Although the cell lines used were not specifically authenticated by us, each showed the expected specific phenotypic characteristics in culture.
Mycoplasma contamination	Cell lines, such as 4T1 cells, and hUVECs were routinely tested for Mycoplasma contamination using the universal mycoplasma detection kit (ATCC). No Mycoplasma contamination was observed in any of the cell lines and hUVECs.
Commonly misidentified lines<br>(See ICLAC register)	No commonly misidentified cell lines were used in the study.

Animals and other research organisms

Policy information about studies involving animals; ARRIVE guidelines recommended for reporting animal research, and Sex and Gender in Research

Laboratory animals	BALB/cByJ (2 and 14 months old), SAMR1 (7 months old), SAMP8 (7-12 months old) and C57BL/6 mice (3 and 15-18 months old).
Wild animals	No wild animals were used in the study.
Reporting on sex	The findings mainly apply to male mice, with the exception of BALB/cByJ female mice bearing breast tumors. Due to the probe being recovered in urine, we had to exclude the sex variability in urinary physiology.
Field-collected samples	No field collected samples were used in the study.
Ethics oversight	All animal procedures were approved by the CIPF and UV Ethics Committees for Research and Animal Welfare (CEBA) and conducted in accordance with the recommendations of the Federation of European Laboratory Animal Science Associations (FELASA).

Note that full information on the approval of the study protocol must also be provided in the manuscript.

Flow Cytometry

Plots

Confirm that:

- [ ] The axis labels state the marker and fluorochrome used (e.g. CD4-FITC).
- [x] The axis scales are clearly visible. Include numbers along axes only for bottom left plot of group (a 'group' is an analysis of identical markers).
- [ ] All plots are contour plots with outliers or pseudocolor plots.
- [x] A numerical value for number of cells or percentage (with statistics) is provided.

Methodology

Sample preparation

Briefly, the right kidney and a portion of liver were extracted and minced for enzymatic dissociation of tissue (collagenase/ dispase and DNAse) in a gentleMACS Octo dissociator (Miltenyi) with heaters. Subsequently, the cells were incubated with CD31 and CD45 antibodies and one portion was used with the WOS-Cy7Gal probe while the other was fixed and permeabilized. The fixed cell suspension was incubated first with the p16 and Lamin B1 primary antibodies and then with the appropiate secondary antibody.

Instrument

SLR-Fortessa X-20 (BD)

Software

FlowJo_v10.8.1 software

Cell population abundance

Concerning the liver of SAMR1 mice, there are approximately 40% CD31+ and CD45+ cells, while in SAMP8 animals these populations comprise about 50% of live cells. Regarding the kidney, there are approximately 15% of CD31+ and CD45+ cells in SAMR1 animals, while in SAMP8 mice these populations comprise about 30%. The percentage of live cells in both tissues was over 90% (using DAPI to discard dead cells). For the analysis of senescence-associated markers, median fluorescence intensity (MFI) was measured, with the exception of lamin B1, where the percentage of cells positive for this marker was assessed in both organs and represented in figure 4.

Gating strategy

First, brain cells were gated by size (FSC) and cellular complexity (SSC), discarding cellular debris. Second, singlets were gated using SCC-H and SCC-A. Next, live or DAPI- cells were selected and CD45+ and CD31+ cells were gated. Finally, all senescence markers were assessed within these two cells populations.

- [ ] Tick this box to confirm that a figure exemplifying the gating strategy is provided in the Supplementary Information.
